# c-IAP1 and c-IAP2 Redundancy Differs between T and B Cells

**DOI:** 10.1371/journal.pone.0066161

**Published:** 2013-06-17

**Authors:** Maria Letizia Giardino Torchia, Dietrich B. Conze, Jonathan D. Ashwell

**Affiliations:** Laboratory of Immune Cell Biology, National Cancer Institute, National Institutes of Health, Bethesda, Maryland, United States of America; Temple University School of Medicine, United States of America

## Abstract

Cellular Inhibitors of Apoptosis 1 and 2 (c-IAP1 and c-IAP2) are ubiquitin protein ligases (E3s) that constitutively ubiquitinate and induce proteasomal-mediated degradation of NF-κB Inducing Kinase (NIK) and repress non-canonical NF-κB activation. Mice expressing an E3-inactive c-IAP2 mutant (c-IAP2^H570A^) have constitutive activation of non-canonical NF-κB, resulting in B cell hyperplasia and T cell costimulation-independence. If, and if so to what extent, c-IAP1 and c-IAP2 are redundant in NF-κB regulation in these mice is not known. Here we have generated mice expressing a mutant c-IAP1 that lacks E3 activity (c-IAP1^H582A^). These mice were phenotypically normal and did not have constitutive NF-κB activation in B cells or MEFs. siRNA-mediated knockdown of c-IAP2 showed that accumulated c-IAP2, resulting from lack of c-IAP1-dependent degradation, compensated for absent c-IAP1 E3 activity. Surprisingly, c-IAP1^H582A^ T cells had a lower p100/p52 ratio than wild type T cells, and in the absence of costimulation proliferated to a degree intermediate between wild type and c-IAP2^H570A^ T cells. Therefore, although c-IAP1 and c-IAP2 both can repress constitutive NF-κB activation, the relative importance of each varies according to cell type.

## Introduction

Inhibitors of apoptosis (IAP) constitute an evolutionarily-conserved family of proteins characterized by the presence of one or more Baculovirus IAP Repeat (BIR) domains, which mediate protein-protein interactions. Among this family, cellular- IAP 1 and 2 (c-IAP1 and c-IAP2) contain a RING domain that confers ubiquitin protein ligase (E3) activity [1]. It was initially thought that c-IAP1 and c-IAP2 inhibit apoptosis by blocking the proteolytic activity of caspases-7 and -9, but subsequent studies have shown that although they bind these caspases they have little inhibitory activity [2]. Consequently, the functional roles ascribed to c-IAPs to date are largely due to their ability to ubiquitinate target proteins, for which they are thought to be largely redundant. One example is their role in tumor necrosis factor α (TNFα) signaling via TNF receptor (TNFR) 1, in which RIP1 is ubiquitinated by c-IAP1- and c-IAP2 [3]–[7]. Furthermore, it was recently shown that c-IAP1 and -2 inhibit the formation of the ripoptosome, presumably by targeting RIP1 for lysine 48 (K48)-linked ubiquitination and degradation by proteasomes [8], [9]. c-IAP1 and c-IAP2 E3 activity has also been implicated in regulating signaling downstream of several pathogen recognition receptors, such as Toll-like receptor (TLR) 4 and retinoic acid-inducible gene I (RIG-I) [10].

Perhaps the most widely appreciated activity of c-IAPs is their role in regulating the activation of the nuclear factor κB (NF-κB) family of transcription factors, which are involved in a wide variety of cellular processes including development, survival, growth, and immune responses. NF-κB family members p50, p52, c-Rel, RelA (p65), and RelB are normally sequestered in the cytosol via their interaction with the ankyrin repeats of inhibitors of NF-κB (IκB) proteins. There are two main mechanisms for NF-κB activation, canonical and non-canonical [11]. The canonical pathway, which is activated by the majority of NF-κB-inducing stimuli, involves phosphorylation of IκBα by the IKKβ subunit of IκB kinase (IKK), followed by IκBα K48-linked ubiquitination and proteasome-mediated degradation. The degradation of IκB frees cytosolic NF-κB heterodimers, which migrate to the nucleus and upregulate transcription of target genes [12]. The non-canonical pathway is downstream of a limited number of receptors, such as CD40, lymphotoxin-β receptor (LTBR), and B-cell activating factor receptor (BAFF-R), which are typically expressed by B cells [13]–[16]. In resting cells, the kinase NIK associates with TRAF-3, which in turn associates with TRAF-2 bound to c-IAP1 or c-IAP2. It is in this inhibitory complex that NIK is constitutively ubiquitinated by c-IAP1 and c-IAP2, making it a target for proteasomal degradation. Upon engagement with ligand this complex is recruited to the receptor where c-IAP1 and c-IAP2 ubiquitinate TRAF2 and TRAF3 instead of NIK, inducing their proteasome-mediated degradation. As a result of being freed from the inhibitory complex NIK levels increase leading to phosphorylation and activation of IKKα. Activated IKKα phosphorylates the NF-κB family member p100, inducing its C-terminal ubiquitination and controlled proteolysis to an active fragment called p52. p52/RelB heterodimers migrate to the nucleus and activate gene transcription [16]. Consistent with this, tandem c-IAP deletions as well as TRAF3 deletions in cell lines derived from multiple myeloma patients have increased non-canonical NF-κB activation [17]–[19]. Furthermore, TRAF2- and TRAF3-deficient B cells have increased levels of p52 [20]–[22].

The function of c-IAP1 and c-IAP2 is often studied by taking advantage of IAP antagonist drugs (SMAC mimetics) that induce degradation of both c-IAP1 and c-IAP2 [4], [23]–[26], making it difficult to dissect possibly distinct roles for the two proteins. Moreover, individual c-IAP1 and c-IAP2 knockout (KO) mice appear to be normal in the unperturbed state [27], [28]. Paradoxically, knock-in mice expressing an E3-inactive mutant of c-IAP2 (c-IAP2^H570A^) have constitutively activated non-canonical NF-κB and a variety of abnormalities, such as enlarged gut associated lymphoid tissue (GALT), marginal zone B cell hyperplasia, increased B cell survival, and hyperproliferative B and T cells [29], [30]. In c-IAP2^H570A^ mice, mutant c-IAP2 protein levels are increased due to lack of autoubiquitination and degradation. Because one TRAF2 trimer can bind only one c-IAP molecule at a time [31], [32], it was proposed that E3-dead c-IAP2 competes with c-IAP1 for TRAF2 binding. Furthermore, co-expression of c-IAP1 and c-IAP2^H570A^ prevents c-IAP1 induced NIK degradation, demonstrating that the lack of E3 activity in c-IAP2 can compromise the tonic repression of c-IAP1 on non-canonical NF-κB [29]. In another report using c-IAP1 and c-IAP2 double-deficient multiple myeloma cells or SMAC mimetics, it was found that c-IAP1 and -2 share the ability to repress the non-canonical NF-κB pathway at the level of the inhibitory complex [33]. The same result was obtained using c-IAP1 and -2 knockout MEFs [34]. More definitive evidence was provided by the analysis of B cells lacking both c-IAP1 and c-IAP2, which had constitutive activation of non-canonical NF-κB whereas the single knockout counterparts did not [35].

The evidence to date has supported the notion that c-IAP1 and c-IAP2 have redundant roles in the regulation of NF-κB, and loss of both is required to see a biological phenotype. In this study we examine mice expressing E3-inactive c-IAP1 and find that unlike in B cells, loss of this activity in T cells cannot be fully replaced by c-IAP2.

## Materials and Methods

### Mice

c-IAP1^H582A^ knock-in mice were generated by homologous recombination using BamH1-Xba1 and Xba1-EcoRV recombination arms that were obtained from BAC DNA (clone 239-13P; Research Genetics). The BamH1-Xba1 arm contained exons 4, 5, 6 and 7, the Xba1-EcoRV arm contained genomic sequence downstream of exon 7, and both were subcloned into shuttle vectors. The H582A mutation and accompanying novel Spe I restriction endonuclease site were then inserted into exon 7 using mutagenic primers and the QuickChange mutagenesis kit from Stratagene. The presence of H582A in exon 7 and wild-type sequence of the remaining exons in the BamH1-Xba1 arm were confirmed by direct sequencing. The recombination arms were subcloned into pLTM260 on either side of an expression cassette containing neomycin cDNA flanked by two LoxP and FRT recombination sites. The resulting targeting vector was linearized, transfected into ES cells, and integration of the c-IAP1 targeting construct at the c-IAP1 locus was determined by Southern blotting and long-range polymerase chain reactions (LR-PCR) on DNA obtained from stable clones. Integration of H582A was determined by LR-PCR (5′-CAGCACAGAGAAAGTAGGAGAGCG-3′ and 5′-GACATACAGCAAGCATCCCAACTC-3′) followed by a Spe1 restriction endonuclease digestion of the PCR products. The clone that correctly integrated the targeting construct at the c-IAP1 locus was then injected into blastocysts and chimeric mice were generated. Germline transmission in the F1 offspring was determined using the Spe1-coupled LR-PCR reaction and mice positive for H582A were backcrossed 6 additional times to the C57BL/6 (B6) background. B6 mice maintained in the same room of the National Cancer Institute vivarium were used as controls. Study protocols were approved by the Institutional Animal Care and Use Committee of the National Cancer Institute.

### Cell Preparation and Purification

B and T cells were purified respectively from spleen or lymph nodes using Easy Sep enrichment kit (StemCell Technologies) following the manufacturer’s instructions, and the number of live cells was assessed by Trypan blue exclusion. Purity was determined by flow cytometry, and for all experiments was >90%. Cells were cultured in RPMI 1640 supplemented with 10% fetal calf serum, 100 U/ml penicillin, 100 µg/ml streptomycin, 2 mM L-glutamine, and 50 µM β-mercaptoethanol (complete medium). MEFs were prepared from day 13.5 embryos as described [36] and maintained in DMEM supplemented as above.

### Reagents and Antibodies

Anti-mouse CD3 (145-2C11), anti-mouse CD28 (37.51), anti-mouse CD40 (HM40-3) and all antibodies for flow cytometry were purchased from BD Biosciences. Anti-β-actin was purchased from Sigma-Aldrich, anti-p100/p52, anti-NIK and anti c-IAP1 were purchased from Cell Signaling, anti-cIAP1/2 from R&D, and anti-IκBα from Santa Cruz. MG-132 was bought from Calbiochem, and chloroquine, Protease Inhibitor Cocktail for use in tissue culture media (including Aprotinin, Bestatin, E-64, Leupeptin, Pepstatin A) and lipopolysaccharide (LPS) were purchased from Sigma.

### Flow Cytometry

Stainings were performed in PBS supplemented with 0.1% FCS and 0.01% NaN_3_ in the presence of 1∶500 Fc-blocking antibody (2.4G2). Flow cytometry was done with a BD LSRII cytometer using BD FACSDiva software (BD Biosciences). Data were analyzed with FlowJo software (TreeStar).

### Proliferation Assay

Assays were performed in 96-well flat-bottomed plates in a final volume of 200 µl. In some experiments, wells were coated with anti-CD3 alone or in combination with anti-CD28 at the indicated concentrations for 1 hr at 37°C or overnight at 4°C in PBS. Cells were cultured for 48 hr, pulsed with 1 µCi [^3^H]-thymidine, and harvested 18 hr later. [^3^H]-thymidine uptake was determined with a Wallac 1450 MicroBeta Liquid Scintillation Counter. All experimental points were performed in triplicate.

### Immunoblotting and siRNA

Cells were lysed in a buffer containing 20 mM Tris pH 7.5, 150 mM NaCl, 1% Triton X-100, 1 mM EDTA, 30 mM NaF, 2 mM sodium pyrophosphate supplemented with Complete protease inhibitor cocktail (Roche), and the detergent-soluble lysate was collected after centrifugation. Lysates were normalized to protein concentration, denatured in sample buffer (50 mM Tris pH 6.8, 10% glycerol, 2% SDS, 2% β- mercaptoethanol, and 0.04% bromophenol blue), resolved by SDS-PAGE, and immunoblotted. For detection of c-IAP1 and p100/p52, cells were normalized to cell number and lysed in sample buffer. Densitometry was performed using the ImageJ gel analysis tool. For knockdown studies, 5×10^5^ MEFs were plated in 6 well plates and 16 h later transfected with 30 nM of Universal Lo GC content non-targeting or c-IAP2 Stealth iRNA siRNA using Lipofectamine RNAiMAX (Invitrogen) following the manufacturer’s protocol.

### ELISA

Supernatants from T cells stimulated with plate-bound anti-CD3 or anti-CD3 plus anti-CD28 were collected at the indicated times and the levels of IFNγ measured using the ELISA Ready-SET-Go! kit (eBioscience) according to the manufacturer’s protocol.

### Real-time PCR

Total RNA was extracted from purified T cells using RNeasy Mini kit (Qiagen) and reverse transcribed using the Superscript II Reverse Transcriptase kit (Invitrogen) following the manufacturer’s protocol. IL-2 and hypoxanthine phosphoribosyltransferase (HPRT) mRNA was quantified using the respective primers, SYBR Green PCR Master Mix (Applied Biosystems), and the 7500 Real Time PCR System (Applied Biosystems). Values were normalized to HPRT and the percent increase relative to wild type (WT) was calculated by dividing c-IAP1^H582A^ values by WT values.

## Results

### Generation of c-IAP1^H582A^ Mice

**Figure 1:**
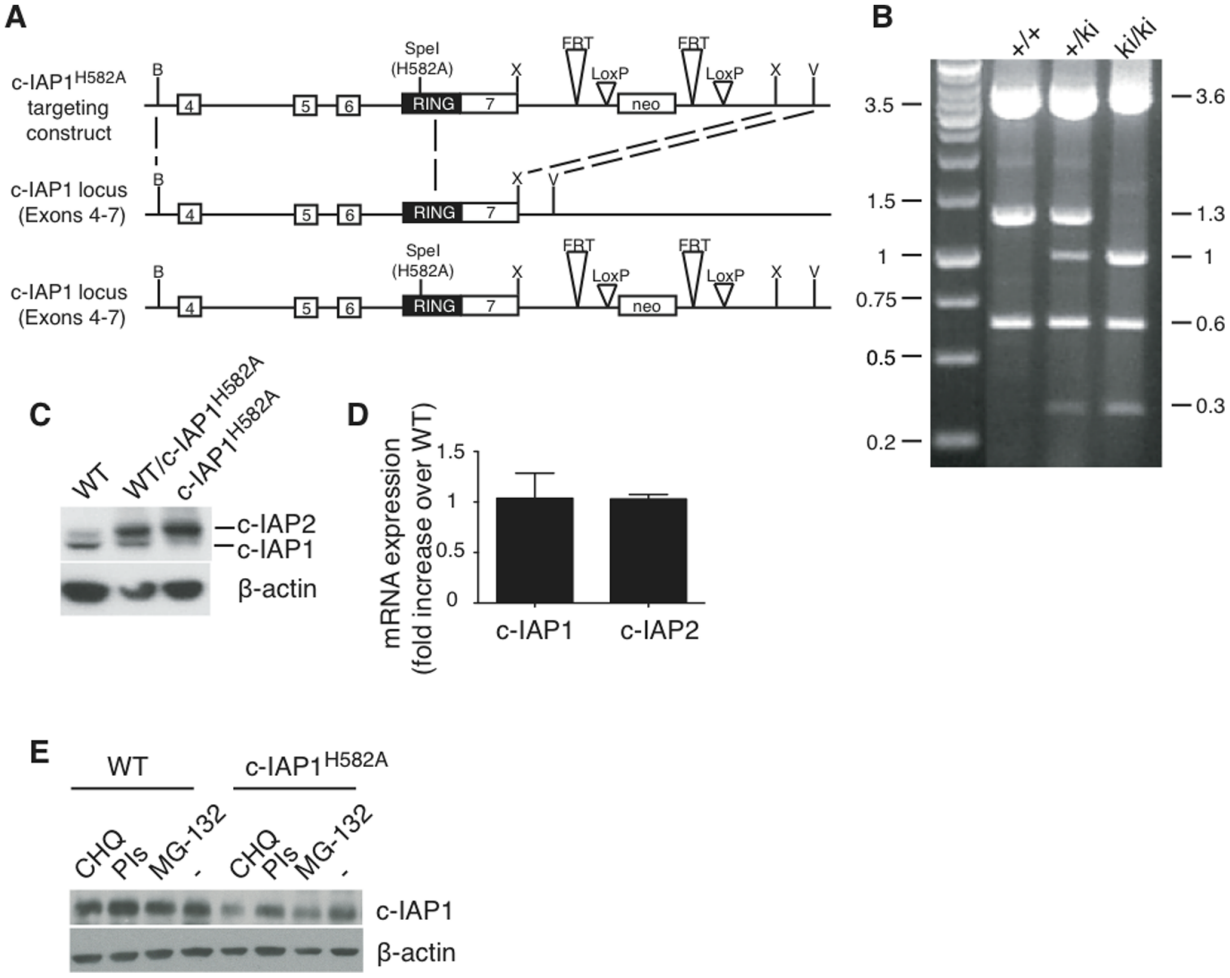
Generation of c-IAP1^H582A^ mice. (A) Schematic representation of the c-IAP1 targeting construct and the recombination strategy. (B) c-IAP1^H582A^ mice were distinguished from WT littermates by LR-PCR and Spe I digestion. (C) c-IAP1 and c-IAP2 expression in splenocytes from WT, c-IAP1^H582A^ heterozygous and homozygous mice were determined by immunoblotting. (D) *Ciap1* and *Ciap2* mRNA expression was determined in WT and c-IAP1^H582A^ splenocytes by real-time PCR. Bars represent the fold increase over WT expression. (E) WT or c-IAP1^H582A^ splenocytes were lysed in sample buffer after 8 hr of incubation with 25 mM chloroquine (CHQ), 1∶200 of the Sigma protease inhibitor cocktail (PIs), 10 µM MG-132, or complete medium (−) and c-IAP1 expression was analyzed by immunoblotting.

**Figure 2:**
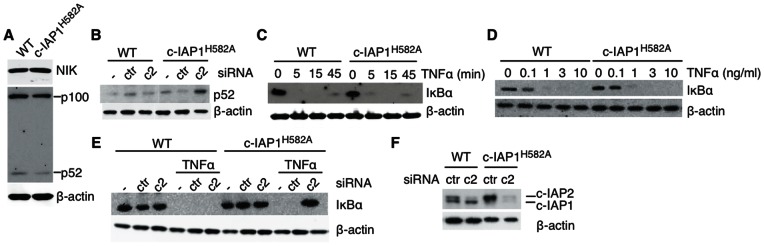
c-IAP2 compensates for c-IAP1^H582A^ in non-canonical NF-κB inhibitory- and TNFR1 complexes. (A) Expression of p100 and p52 in MEFs was detected by immunoblotting. (B and F) MEFs were untreated (−) or transfected with non-targeting control (ctr) or c-IAP2 siRNA (c2) for 24 hr and lysed. p52 and c-IAP1/2 levels were determined by immunoblotting. IκBα expression was analyzed by immunoblotting of lysates of MEFs treated for the indicated times with 1 ng/ml of TNFα (C) or 15′ with the indicated doses of TNFα (D). (F) MEFs were untreated (-) or transfected with non-targeting control (ctr) or c-IAP2 siRNA for 24 hr, then treated with 1 ng/ml TNFα for 15′ and lysed. IκBα expression was analyzed by immunoblotting. Lanes were rearranged for clarity.

To understand the function of c-IAP1 E3 activity in vivo, we generated gene-targeted mice in which endogenous c-IAP1 was replaced with an E3-inactive mutant of c-IAP1 (c-IAP1^H582A^) (Figure 1A). A novel yet silent Spe1 restriction endonuclease site was inserted into the c-IAP1 mutant locus to allow for screening of ES cells and mice for the presence of H582A. ES cells that had stably integrated the targeting vector at the c-IAP1 locus were used to generate chimeric mice, which were bred onto the B6 background. The presence of the H582A substitution in F1 offspring and subsequent generations was assessed by long-template PCR (LR-PCR) followed by Spe 1 restriction endonuclease digestion (Figure 1B). As expected, Spe1 digestion of the PCR products from wild-type mice resulted in 3.6, 1.3, and 0.6 Kb bands and introduction of the mutant allele in c-IAP1^+/H582A^ and c-IAP1^H582A/H582A^ mice resulted in the gene dose-dependent disappearance of the 1.3 Kb and appearance of the 0.3 and 1 Kb bands. Immunoblotting of splenocyte lysates using an antiserum recognizing both c-IAP1 and c-IAP2 [29], [37] showed a modest and reproducible reduction of c-IAP1 protein that seemed to be dependent on the lack of c-IAP1 E3 activity, because it was less pronounced in cells heterozygous for the mutant c-IAP1, and a substantial increase in c-IAP2 expression, which was consistent with the role of c-IAP1 in constitutive c-IAP2 ubiquitination and degradation (Figure 1C). Indeed, the increase was due to post-transcription events, as RT-PCR showed that the level of c-IAP2 mRNA in c-IAP1^H582A^ splenocytes was comparable to WT cells (Figure 1D). However, the reduction in c-IAP1 protein was not due to ubiquitination by the highly expressed c-IAP2, because neither proteasome inhibitors nor c-IAP2 silencing in c-IAP1^H582A^ MEFs restored the levels to normal (Figure 1E, 2F and data not shown). Incubation with the lysosomal inhibitor chloroquine or protease inhibitors also did not rescue c-IAP1^H582A^ levels (Figure 1E). Thus, c-IAP1 E3 activity is involved in maintaining physiologic levels of both itself and c-IAP2.

### c-IAP2 Compensates for c-IAP1^H582A^ in Non-canonical NF-κB Inhibitory- and TNFR1 Complexes

To determine if the increased expression of c-IAP2 in c-IAP1^H582A^ cells (Figure 1C) compensated for the loss of c-IAP1 E3 activity, we analyzed NF-κB activation in MEFs.Western blot analysis of c-IAP1^H582A^ MEFs did not show increases in NIK levels or abnormal processing of p100 to p52 (Figure 2A). To analyze the contribution of c-IAP1 E3-activity to the constitutive repression of non-canonical NF-κB, we silenced c-IAP2 in WT or c-IAP1^H582A^ MEFs (Figure 2B and F). p52 expression increased when c-IAP2 was knocked-down in c-IAP1^H582A^ but not WT MEFs, confirming that the E3 activity of c-IAP1 and c-IAP2 is redundant in these cells. In addition to non-canonical NF-κB, c-IAPs play important roles in TNFR1 signaling [1]. Ligation of TNFR1 induces the formation of a signaling complex that includes c-IAP1, c-IAP2, TRAF2, TRAF5, and RIP1 [38]. RIP1 ubiquitination by c-IAP1 and c-IAP2 facilitates the recruitment and activation of TAK1 and IKK complexes by binding to TAB2 and NEMO, respectively [38]. Given that c-IAP E3 activity is required for TNFα dependent RIP1 ubiquitination [5], [39], we asked if TNFR1 signaling was intact in c-IAP1^H582A^ cells. The level of IκBα was the same in both WT and c-IAP1^H582A^ cells, as was the kinetics and degree of degradation after TNFα stimulation (Figure 2C and D). Whereas knockdown of c-IAP2 in WT MEFs did not affect TNF-induced IκBα degradation, it abrogated IκBα degradation in c-IAP1^H582A^ MEFs (Figure 2E and F), unmasking the effect of c-IAP1 E3 loss and confirming that in this complex the E3 activities of c-IAP1 and c-IAP2 are redundant. These results indicate that c-IAP1 ubiquitin protein ligase activity *per se* is required for the control of the non-canonical NF-κB and TNFR1 signaling pathways, but its effects in vivo are compensated for by the high level of E3-active c-IAP2.

### Normal B Cell Cellularity and Function in c-IAP1^H582A^ Mice

**Figure 3:**
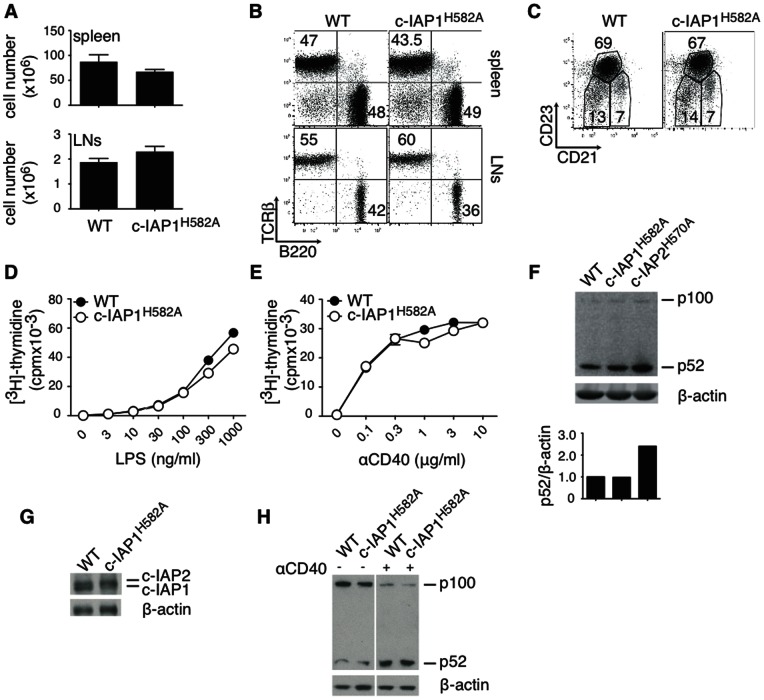
Normal B cell cellularity and function in c-IAP1^H582A^ mice. (A) Cellularity of spleen and pooled lymph nodes (axial, brachial, superficial cervical and inguinal) from 3 WT and 3 mutant mice. (B) B and T cell distribution was analyzed by flow cytometry. (C) Distribution of marginal zone (CD21^hi^CD23^−^), follicular (CD21^int^CD23^hi^), and immature (CD21^−^CD23^−^) B cells in spleens from WT and c-IAP1^H582A^ mice were analyzed by flow cytometry. (D and E) Purified B cells were cultured in vitro with the indicated concentrations of LPS or anti-CD40 for 48 hr, pulsed with ^3^H-thymidine, and harvested 18 hr later. (F) Expression of p100 and p52 in purified B cells was detected by immunoblotting. For each sample densitometry of p52 was performed and the results expressed as its ratio to β-actin. (G) c-IAP1/2 expression in freshly purified B cells. (H) Immunoblot of B cells freshly purified or stimulated for 8 hr with 1 µg/ml anti-CD40.

Mice carrying the homozygous c-IAP1^H582A^ mutation were viable and fertile and did not display obvious phenotypic abnormalities. However, to determine whether or not the absence of c-IAP1 E3 activity affected B cell homeostasis as was seen in mice lacking c-IAP2 E3 activity [29], the cellularity and distribution of T and B cells in the spleen and lymph nodes were assessed by flow cytometry. The cellularity of c-IAP1^H582A^ spleen and pooled lymph nodes was comparable to that of WT littermates (Figure 3A), the ratio between B and T cells was normal (Figure 3B), and no differences were detected in the percentage of B cells with marginal zone (CD21^hi^CD23^−^), follicular (CD21^int^CD23^hi^), or immature (CD21^−^CD23^−^) characteristics (Figure 3C). B cells lacking c-IAP2 E3 activity are hyper-responsive to mitogenic stimuli [29]. However, B cells from c-IAP1^H582A^ animals proliferated normally when stimulated with lipopolysaccharide (LPS) or anti-CD40 (Fig. 3D and E). Lack of c-IAP2 E3 activity was also reflected in constitutive activation of non-canonical NF-κB, with constitutive cleavage of the NF-κB family member p100 and high levels of p52 at rest ([29] and Fig. 3F). Western blot analysis of freshly purified c-IAP1^H582A^ B cells showed no alteration in p52 levels (Figure 3F) and, as expected, higher levels of c-IAP2 (Figure 3G). When stimulated with anti-CD40, c-IAP1^H582A^ B cells increased the level of p52 similarly to WT cells (Figure 3H), showing that non-canonical NF-κB regulation is unaffected not only at steady state but also during activation. Therefore, unlike c-IAP2, loss of c-IAP1 ubiquitin protein ligase activity had no obvious effect on non-canonical NF-κB pathway in B cells.

### c-IAP1 and c-IAP2 are only Partially Redundant in T Cells

**Figure 4:**
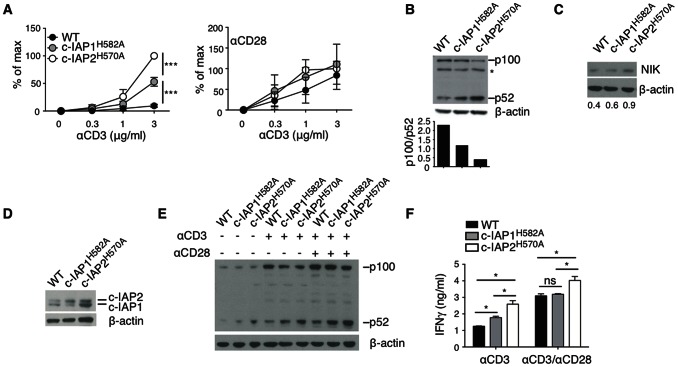
c-IAP1 and c-IAP2 are only partially redundant in T cells. (A) Purified T cells were cultured in vitro with the indicated amount of anti-CD3 in the absence (left panel) or presence (right panel) of 2 µg/ml of anti-CD28. After 48 hr, cells were pulsed with ^3^H-thymidine and harvested 18 hr later. The panel represents the average of four independent experiments and the error bars are the SEM. ***p<0.005. (B) Expression of p100 and p52 in purified T cells was detected by immunoblotting. For each sample densitometry of p100 and p52 was performed and the results expressed as the ratio between each protein and β-actin. Expression of NIK (C) and c-IAP1/2 (D) in purified T cells was detected by immunoblotting. Values in C represent the ratio between NIK and β-actin. (E) p100/52 immunoblot of T cells freshly purified or stimulated for 24 hr with 1 µg/ml anti-CD3±2 µg/ml anti-CD28. (F) T cells were stimulated as in (E) and ELISA was performed on supernatants collected after 24 hr. One of two independent experiments is shown and the error bars are the SD of the duplicates. *p<0.05.

T cells from c-IAP2 E3-dead mice are costimulation-independent [30]. To ask whether the lack of c-IAP1 E3-activity had the same effect, we monitored the proliferation of c-IAP1^H582A^ T cells stimulated with increasing concentrations of anti-CD3 in presence or absence of agonistic anti-CD28 antibodies to provide costimulation. Surprisingly, c-IAP1^H582A^ T cells were able to proliferate in response to TCR engagement alone, although to a lesser extent than c-IAP2^H570A^ T cells, whereas no difference was detected if CD28 was engaged (Figure 4A). Costimulation-independence in c-IAP2^H570A^ T cells is the result of an imbalance between NF-κB subunits p100 and p52 [30]. Western blot analysis of resting c-IAP1^H582A^ T cells showed that the level of p100 was decreased and the level of p52 increased, resulting in a lower ratio between the two (Figure 4B). Activation of non-canonical NF-κB was also reflected in slightly elevated expression of NIK at rest (Figure 4C) which was intermediate between WT and c-IAP2^H570A^ T cells and proportional to the degree of costimulation-independence. Incomplete redundancy in c-IAP1^H582A^ T cells was not due to lack of c-IAP2 up-regulation, as confirmed by immunoblot analysis (Figure 4D). Constitutive activation of NF-κB resulted in approximately a 2.5-fold increase in the NF-κB target genes *Ikba* and *Ciap2* [29] as shown by RT-PCR analysis of freshly purified T cells (data not show). TCR activation increases p52 levels as a result of enhanced p100 synthesis [40]. p52 was higher in c-IAP1^H582A^ T cells than in their WT counterparts after anti-CD3 stimulation and even more so when anti-CD28 was present, but in both cases lower than the level found in c-IAP2^H570A^ T cells (Figure 4E). c-IAP1^H582A^ T cells produced more IFNγ, an NF-κB -responsive gene product [41], than WT T cells when stimulated with anti-CD3 (but less than c-IAP2^H570A^ T cells), whereas no difference was found when anti-CD28 was present (Figure 4F). Thus, the redundancy of c-IAP1 and c-IAP2 in suppressing non-canonical NF-κB signaling is cell-type specific, and c-IAP2 is unable to fully compensate for the lack of c-IAP1 E3 activity in T cells.

## Discussion

Given the high degree of homology between c-IAP1 and c-IAP2, it is not surprising that their functions have often been found to be redundant [42]. The best evidence for this comes from single c-IAP1- and c-IAP2-null mice, which despite the many pathways in which c-IAPs are involved are almost normal [1], [27], [28], whereas deficiency of both is embryonically lethal [7]. A paradigmatic example of c-IAP redundancy in a single cell-type is provided by the analysis of B cell subpopulations in genetically modified mice. Lack of either c-IAP1 or c-IAP2 does not affect the number or distribution of splenic B cell populations, or the extent of p100 processing. On the other hand, deficiency of both is associated with splenomegaly, accumulation of marginal zone (MZ) B cells, and high levels of p52 at rest [35]. Accumulation of MZ B cells and constitutive activation of non-canonical NF-κB was also described in mice with germ-line inactivation of c-IAP2 E3 activity due to a dominant negative effect on c-IAP1 [29]. In the present report we show that lack of c-IAP1 E3 activity does not affect B cell distribution or levels of p52 at rest, likely because of a compensatory effect of WT c-IAP2. Therefore, in B cells c-IAP1 and c-IAP2 regulate non-canonical NF-κB and development in a redundant fashion.

c-IAP1 and c-IAP2 control their own expression posttranscriptionally by autoubiquitination and resulting proteasomal degradation [1]. Additionally, c-IAP1 constitutively ubiquitinates c-IAP2, which accounts for the high level of c-IAP2 in cells from c-IAP1^−/−^ mice [27]. The lack of E3 activity in c-IAP2^H570A^ mice has no effect on c-IAP1 expression, but results in accumulation of mutant c-IAP2 and abnormal NF-κB function [29]. One might expect that lack of cIAP1 E3 activity would result in its accumulation as well, especially because c-IAP2 does not trans-ubiquitinate c-IAP1. However, this is not the case. Indeed, although there was accumulation of c-IAP2, c-IAP1 levels were actually diminished. Because the mRNA levels were normal, this suggests an additional level of posttranscriptional regulation. As we were unable to restore normal levels with protease inhibitors, the mechanism for decreased c-IAP1^H582A^ remains unknown and will require further characterization.

Although in many cases c-IAP1 and c-IAP2 have been found to be redundant, there are examples where unique roles have been ascribed. For example, stimulation via TNFR2 activated c-IAP1, not c-IAP2, resulting in ubiquitination and degradation of TRAF2 and ASK-1 and limiting the duration of signaling [43], [44]. Moreover, c-IAP1^−/−^ mice are highly susceptible to *Chlamydophila pneumoniae* and are resistant to endotoxin-induced shock, likely because of decreased NO and TNF-α production by peritoneal macrophages [45]. c-IAP2^−/−^ mice are more resistant to septic shock, presumably because their peritoneal macrophages undergo apoptosis and thus produce less TNF-α and IL-1β after LPS stimulation [28]. The results in the present report provide a somewhat different example of non-redundant behavior in that it is tissue-dependent, being found in T but not B cells and MEFs. Notably, c-IAP2 was unable to compensate for c-IAP1 in T cells even though its levels were markedly increased. Although the reasons for this are unknown, it is possible that the E3-dead c-IAP1 mutant binds other components of the signaling machinery with higher affinity than c-IAP2, acting in a dominant negative fashion.

Given that targeting of c-IAP1 and c-IAP2 together is being pursued in anti-cancer therapies [46], it is important to understand which of their biological functions are redundant and which are family-member specific. The observations in this study add the caveat that the relative importance of c-IAP1 and c-IAP2 in the same pathway may differ between different cell types.
